# PolB1 Is Sufficient for DNA Replication and Repair Under Normal Growth Conditions in the Extremely Thermophilic Crenarchaeon *Sulfolobus acidocaldarius*

**DOI:** 10.3389/fmicb.2020.613375

**Published:** 2020-12-23

**Authors:** Hiroka Miyabayashi, Rupal Jain, Shoji Suzuki, Dennis W. Grogan, Norio Kurosawa

**Affiliations:** ^1^Department of Environmental Engineering for Symbiosis, Graduate School of Science and Engineering, Soka University, Tokyo, Japan; ^2^Department of Biological Sciences, University of Cincinnati, Cincinnati, OH, United States

**Keywords:** hyperthermophilic archaea, *Sulfolobus acidocaldarius*, DNA polymerase, DNA replication, DNA repair

## Abstract

The thermophilic crenarchaeon *Sulfolobus acidocaldarius* has four DNA polymerases (DNAPs): PolB1, PolB2, PolB3, and Dbh (PolY). Previous *in vitro* studies suggested that PolB1 is the main replicative DNAP of *Sulfolobales* whereas PolB2 and Y-family polymerases Dpo4 (*Saccharolobus solfataricus*) or Dbh are involved in DNA repair and translesion DNA synthesis. On the other hand, there are various opinions about the role of PolB3, which remains to be clearly resolved. In order to examine the roles of the DNAPs of *S. acidocaldarius* through *in vivo* experiments, we constructed *polB2*, *polB3*, and *dbh* deletion strains and characterized their phenotypes. Efforts to construct a *polB1* deletion strain were not successful; in contrast, it was possible to isolate triple gene-deletion strains lacking *polB2*, *polB3*, and *dbh*. The growth of these strains was nearly the same as that of the parent strains under normal growth conditions. The *polB2*, *polB3*, and *dbh* single-deletion strains were sensitive to some types of DNA-damaging treatments, but exhibited normal sensitivity to UV irradiation and several other damaging treatments. Overall, the genotype which exhibited the greatest sensitivity to the DNA-damaging treatments we tested was the Δ*polB2* Δ*polB3* combination, providing the first evidence of overlapping function for these two DNAPs *in vivo*. The results of our study strongly suggest that PolB1 is responsible for the DNA replication of both the leading and lagging strands and is sufficient to complete the repair of most DNA damage under normal growth conditions in *S. acidocaldarius*.

## Introduction

For the inheritance of genetic information from parent to offspring, DNA must be rapidly and accurately replicated. However, DNA damage is always generated due to endogenous and exogenous factors. Unrepaired DNA damage becomes a source of mutations, or leads to cell death in serious situations. Study of bacteria and eukaryotes has revealed various mechanisms that act to preserve genome integrity in the face of DNA damage. Some of these mechanisms, such as photoreversal, correct particular lesions directly while others, such as translesion DNA synthesis (TLS), allow sites of damage to be replicated. Most of the remaining strategies involve repair, which requires enzymatic removal of the damaged site and re-synthesis of the removed portion of the strand. Examples of these latter mechanisms include (i) base excision repair (BER), which removes bases damaged by deamination, alkylation, or oxidation, (ii) nucleotide excision repair (NER), which removes bulky or helix-distorting DNA lesions, (iii) mismatch repair (MMR), which removes misincorporated bases, and double-strand break repair (DSBR), which involves resection of the 5′ ends (Friedberg et al., 2006).

DNA polymerases (DNAPs) play central roles in DNA replication, repair, and recombination, and can be broadly categorized into two functional types: (1) replicative polymerases and (2) accessory polymerases. Replicative polymerases are generally highly processive and accurate, exhibiting 3′ to 5′ exonuclease activity (Johansson and Dixon, 2013). Accessory polymerases are typically non-processive and participate in DNA repair and tolerance pathways (Fuchs and Fujii, 2013). In evolutionary terms, DNAPs are divided into seven families: A, B, C, D, E, X, and Y (Braithwaite and Ito, 1993; Cann and Ishino, 1999; Ohmori et al., 2001; Lipps et al., 2003; Yamtich and Sweasy, 2010). Eukarya employ B-family polymerases as their replicative DNAPs, while bacteria employ C-family polymerases. Within the Archaea, Euryarchaea have a D-family polymerase, which is essential in some species (Cubonová et al., 2013) and at least one B-family polymerase (Makarova et al., 2014; Sarmiento et al., 2014; Cooper, 2018). In contrast, Crenarchaea lack D-family polymerases, whereas they have at least two B-family polymerases; thus, it seems that they employ B-family polymerases but not D-family polymerases as replicative polymerases (Makarova et al., 2014; Sarmiento et al., 2014; Cooper, 2018).

*In vitro*, chain extension by replicative DNAPs is blocked by a variety of template lesions, and this property is consistent with the accuracy required of DNA replication. *In vivo*, however, several mechanisms can overcome the initial blocking effects of template lesions, thereby allowing the affected region of DNA to be replicated (White, 2007; Aguilera and Gómez-González, 2008; Grogan, 2015). In one mechanism, a translesion DNAP temporarily replaces the replicative DNAP and synthesizes a short tract of DNA on the strand opposite DNA damage (Ohmori et al., 2001; White, 2007; Yang and Gao, 2018). In general, this property of TLS is exhibited by Y-family DNAPs (Ohmori et al., 2001). In Eukarya these TLS DNAPs include Polη, Polι, Polκ, and Rev1 whereas bacterial examples include PolIV and PolV, and archaea have Dbh and Dpo4 (Ohmori et al., 2001; Boudsocq et al., 2004; Sakofsky et al., 2012; Vaisman and Woodgate, 2017). All bacteria and eukarya have a Y-family DNAP, but less than half of archaea encode one (Kelman and White, 2005; Jozwiakowski et al., 2015; Cooper, 2018). This raises the question as to whether other DNAPs substitute for Y-family polymerases in many archaea. Conversely, in particular eukarya and bacteria, one or more B-family polymerases have been found to participate in TLS processes in ways that appear to complement the TLS functions of Y-family polymerases (Yang and Gao, 2018). It remains unclear whether this latter situation occurs in Archaea, however.

Many archaea inhabit extreme environments in which the conditions tend to promote DNA damage (Lindahl, 1993; White and Allers, 2018), and as a group, Archaea seem to exhibit robust DNA repair (White and Allers, 2018). Archaeal DNA information-processing enzymes are generally homologous to those of eukarya rather than those of bacteria (Kelman and White, 2005), and this pattern applies to known and putative DNA repair proteins of archaea (White and Allers, 2018). However, homologues of certain proteins required for specific pathways of DNA repair are lacking from major groups of archaea (Kelman and White, 2005), which implies that the functional details of the corresponding processes in these archaea are not yet completely understood.

The thermophilic crenarchaeon *Sulfolobus acidocaldarius* has four DNAPs: PolB1, PolB2, PolB3, and Dbh. Dbh belongs to the Y family of polymerases and is represented by Dpo4 in *Saccharolobus solfataricus* (Boudsocq et al., 2004). Previous *in vitro* studies of DNAPs of *Sulfolobales* by Choi et al. (2011) and Peng et al. (2016) indicate that PolB1 and Dpo4 exhibit high DNAP activity, whereas PolB2 and PolB3 are relatively inefficient polymerases. Consistent with other B-family polymerases, PolB1, PolB2, and PolB3 have 3′-to-5′ exonuclease activity, which is lacking in Dbh and Dpo4. Bauer et al. (2012) reported that PolB3 of *S. solfataricus* has moderate DNAP activity, moderate accuracy, and relatively low affinity for DNA template. The Y-family polymerases of *Sulfolobales* Dpo4 and Dbh can bypass UV photoproducts, deaminated bases, oxidized bases, methylated bases, and apurinic/apyrimidinic sites (AP sites) *in vitro*, although with differences in efficiency (Boudsocq et al., 2004; Choi et al., 2011; Peng et al., 2016). Similarly, PolB2 and PolB3 has been reported to bypass some DNA damage (i.e., hypoxanthine and 8-oxoG) *in vitro* (Choi et al., 2011; Table 1). In addition, PolB2 can bypass uracil and methylate bases, and PolB3 can bypass cyclobutane thymine dimers (Table 1), and the expression level of *polB2* has been found to increase after UV irradiation (Fröls et al., 2007; Götz et al., 2007; Feng et al., 2020).

**TABLE 1:** Translesion synthesis activities *in vitro* reported for DNAPs of Sulfolobales*.

Damage**	PolB1	PolB2	PolB3	PolY***
Hypoxanthine	−	+	+	+
Uracil	−	+	−	+
8-oxoG	−	+	+	+
AP site	+	−	−	+
N^2^-MeG	+	+	−	+
O^6^-MeG	+	+	−	+
N^2^-BzG	−	−	−	+
O^6^-BzG	−	−	−	+
CTD	−	−	+	+

*Data are for Dpo4 (*S. solfataricus* and related isolates) (Boudsocq et al., 2001; Choi et al., 2011; Peng et al., 2016).

**8-oxoG, 8-oxo-7,8-dihydrodeoxyguanosine; AP, apurinic; Me, methyl; Bz, benzyl; CTD, cis-syn cyclobutane thymine (dimer); + indicates translesion synthesis ability and − indicates no translesion synthesis ability in vitro.

***Although Dbh shares some of the properties of Dpo4, it has been found to be less processive than Dpo4 and inefficient at bypass of AP and CTD sites (Boudsocq et al., 2004).

A PolB1 is found in all members of the TACK (Thaumarchaota, Aigarchaota, Crenarchaeota, and Korarchaeota) superphylum of Archaea (Makarova et al., 2014; Cooper, 2018). Examples of PolB2 are scattered among Crenarchaeota, Euryarchaea, and Aigarchaota (Makarova et al., 2014; Cooper, 2018), and PolB3 is found in almost all archaea except Thaumarchaota (Makarova et al., 2014; Cooper, 2018). Previous *in vivo*, *in vitro*, and bioinformatic studies suggest that PolB1 is the main replicative DNAP in these archaea (Choi et al., 2011; Makarova et al., 2014). The biochemical evidence that PolB2 and Dbh may be specialized for DNA repair, TLS, or both (Boudsocq et al., 2004; Choi et al., 2011) has been supported by phenotypic analysis of *dbh* (*polY*) mutants of *S. acidocaldarius* (Sakofsky et al., 2012; Jain et al., 2020), and *dpo2* (*polB2*) mutants of a related genus (Feng et al., 2020). Although the PolB3 class of polymerases has the widest distribution among archaea (Cooper, 2018), its functional significance remains unclear.

In order to examine the roles of the DNAPs of *S. acidocaldarius* systematically through *in vivo* experiments, we sought to construct strains completely lacking *polB1*, *polB2*, *polB3*, and *dbh* (*polY*) genes, and characterized their mutant phenotypes, examining sensitivity to UV irradiation, DNA-damaging agents, heat-shock, and DNA replication inhibitors.

## Materials and Methods

### Strains and Growth Conditions

The strains used in this study are listed in Table 2. The growth conditions were previously reported (Suzuki and Kurosawa, 2017). The *S. acidocaldarius* pyrimidine-auxotrophic, restriction endonuclease *Sua*I-deficient and DNA photolyase Phr-deficient strain DP-1 (Δ*pyrE* Δ*suaI*Δ*phr*) was used as the parent strain (Suzuki and Kurosawa, 2016, 2017) for constructs HM1–HM7. Triple-polymerase mutants RJ11 and RJ1101 were constructed using *S. acidocaldarius* uracil auxotroph MR31 and remain SuaI^+^ Phr^+^. *S. acidocaldarius* strains were cultivated in xylose and tryptone (XT) medium (pH 3) (Grogan, 1995) containing 1× basal salts, 20 μL of trace mineral solution, 2 g/L xylose, and 1 g/L tryptone in 1 L Milli-Q H_2_O at 75°C with or without shaking (160 rpm). To solidify plates, identical components of 1× basal salts containing 2.9 g MgSO_4_⋅7 H_2_O and 0.5 g CaCl_2_⋅2H_2_O were used. For growth of the uracil (pyrimidine)-auxotrophic strain, 0.02 g/L uracil was added to XT medium (XTU). The XTU medium was supplemented with 50 μg/mL 5-fluoro-orotic acid (FOA) (XTUF) for counterselection in the pop-out recombination method.

**TABLE 2:** Strains and DNA sequences used in this study.

Strains or DNAs	Relevant characteristic(s)	Source or references
**Strains**		
***S. acidocaldarius***		
DP-1	SK-1 with Δ*phr* (Δ*pyrE* Δ*suaI* Δ*phr*)	Suzuki and Kurosawa, 2016, 2017
HM-1	DP-1 with Δ*polB2* (Δ*pyrE* Δ*suaI* Δ*phr* Δ*polB2*)	This study
HM-2	DP-1 with Δ*polB3* (Δ*pyrE* Δ*suaI* Δ*phr* Δ*polB3*)	This study
HM-3	DP-1 with Δ*dbh* (Δ*pyrE* Δ*suaI* Δ*phr* Δ*dbh*)	This study
HM-4	HM-1 with Δ*polB3* (Δ*pyrE* Δ*suaI* Δ*phr* Δ*polB2*Δ*polB3*)	This study
HM-5	HM-1 with Δ*dbh* (Δ*pyrE* Δ*suaI* Δ*phr*Δ*polB2*Δ*dbh*)	This study
HM-6	HM-2 with Δ*dbh* (Δ*pyrE* Δ*suaI* Δ*phr* Δ*polB3*Δ*dbh*)	This study
HM-7	HM-5 with Δ*polB3* (Δ*pyrE* Δ*suaI* Δ*phr* Δ*polB2*Δ*polB3*Δ*dbh*)	This study
MR31	*pyrE131*	Reilly and Grogan, 2001
RJ11	*pyrE131*, ΔSaci_0074, ΔSaci_0554, ΔSaci_2156	This study
RJ12	MR31 Pyr+	Jain et al., 2020
RJ1101	RJ11 with restored *pyrE* (ΔSaci_0074, ΔSaci_0554, ΔSaci_2156)	This study
**Plasmid DNA**		
placSpyrE	Plasmid DNA carrying 0.8 kb of the 5′ and 3′ homologous regions of the *suaI* locus at both ends of the *pyrE*-*lacS* dual marker	Suzuki and Kurosawa, 2017
pStoCassV4	Kan^*R*^ pUC19 derivative carrying engineered *Sulfurisphaera tokodaii pyrE* gene	Jain et al., 2020
**PCR products**		
MONSTER-polB1	Linear DNA containing the 39-bp 5′ and 30-bp 3′ sequences of the *polB1* flanking regions and a 39-bp region of *polB1* as the Tg-arm at both ends of the *pyrE*-*lacS* dual marker	This study
MONSTER-polB2	Linear DNA containing the 39-bp 5′ and 30-bp 3′ sequences of the *polB2* flanking regions and a 39-bp region of *polB2* as the Tg-arm at both ends of the *pyrE*-*lacS* dual marker	This study
MONSTER-polB3	Linear DNA containing the 39-bp 5′ and 30-bp 3′ sequences of the *polB3* flanking regions and a 39-bp region of *polB3* as the Tg-arm at both ends of the *pyrE*-*lacS* dual marker	This study
MONSTER-polY (Dbh)	Linear DNA containing the 39-bp 5′ and 30-bp 3′ sequences of the *dbh* flanking regions and a 39-bp region of *dbh* as the Tg-arm at both ends of the *pyrE*-*lacS* dual marker	This study

### General DNA Manipulation

The reagents used in these experiments were prepared as previously described (Suzuki and Kurosawa, 2017). PCR products were purified using the NucleoSpin Gel and PCR Clean-up kit (Macherey-Nagel) or Microcon-100 centrifugal concentrators.

### Construction of Knockout Cassettes

The plasmids and DNAs used in this study are shown in Table 2, and the PCR primers used in this study are listed in Table 3. The multiple gene knockout system with one-step PCR (MONSTER) was used to prepare *polB1* (Saci_1537), *polB2* (Saci_2156), *polB3* (Saci_0074), and *dbh* (*polY*) (Saci_0554) knockout cassettes (MONSTER-polB1, MONSTER-polB2, MONSTER-polB3, and MONSTER-polY, respectively) and to construct *polB1*, *polB2*, *polB3*, and *dbh* (*polY*) deletion strains (Suzuki and Kurosawa, 2017). In brief, the MONSTER-polx (*x* = B1, B2, B3, or Y) cassettes were amplified from placSpyrE as a template using the MONSTER-polx-F/R primers (containing 39-bp 5′ and 30-bp 3′ sequences of the *polx* flanking region and a 39-bp region of *polx* (*x* = *B1*, *B2*, *B3*, or *Y*) as the target gene (Tg)-arm at the 5′ ends of the primers) and Emerald Amp MAX PCR Master mix (Takara Bio) under the following conditions: 94°C for 3 min; 30 cycles of 94°C for 30 s, 50°C for 30 s, and 72°C for 3 min; and a final extension at 72°C for 3 min. The purified PCR products (200 ng/μL in 5 mM Tris–HCl, pH 8.5) were used for subsequent electrotransformation. Production of targeted *pyrE* cassettes and replacement of the Saci_0074, Saci_0554, and Saci_2156 genes followed the scheme described previously for the Saci_0554 and Saci_1367 genes (Jain et al., 2020).

**TABLE 3:** Primers used in this study.

Designation	Sequence (5′-3′)
HM constructs*	
MONSTER-polB1-F	aagtttatatcgtaattctacttaatttatatattgtat**ataatagaagggagagttttaaattaaaat**GTTTTTCTCTATATCAATCTC
MONSTER-polB1-R	ctttatggaaaaatcaaagagtgttgcttgtttggacatCTCCTAGATCTAAAACTAAAG
MONSTER-polB2-F	tagctgaaggtgcttgtaatggaaggctatctcgttgat**taggataatacaaagagatgagatagttga**GTTTTTCTCTATATCAATCTC
MONSTER-polB2-R	atctaagactaggactacactgttgtaagacggtaaagcCTCCTAGATCTAAAACTAAAG
MONSTER-polB3-F	tgagtaaatattttatttagtttctagtagataatcagt**gtgcattaaaagttctggggttatttgggg**GTTTTTCTCTATATCAATCTC
MONSTER-polB3-R	atcatatgaaaagtctaatacaaagaaatcctctaacacCTCCTAGATCTAAAACTAAAG
MONSTER-polY-F	gccttaaatgcttatacaccaaatactaaatgtaaatga**ggagtaaaattagataacataataatcaat**GTTTTTCTCTATATCAATCTC
MONSTER-polY-R	cttgtgcgaagaaataatcaaaatcaacgaatatcactaCTCCTAGATCTAAAACTAAAG
polB1-out-F	atcagtaattataagtaatctac
polB1-out-R	aagattatgcaaaacaagtaac
polB2-out-F	gctttatacgatgaagtgac
polB2-out-R	ctctgaaatattctcttaaatc
polB3-out-F	ctatcaatttctatataaagaac
polB3-out-R	caaaaacataaaaatgctaatag
polY-out-F	gcactaaaagtaatgagaatag
polY-out-R	gaggttttataaattacgtttac
polB1-in-1	tactataacaattacgtatacg
polB1-in-2	cagtgagaatatatgcttttg
polB1-in-3	gctacattatgtgtgtatgg
polB2-in-1	tgcctgtgacaaggttaaag
polB3-in-1	gaaactgtatttgattaaagac
polB3-in-2	tcaggacacagaattgaatg
**RJ constructs**	
delStc Saci0074Fwd2	TATTTCTACGCTGTGGTAGATGATAGAGAAGATGTATCTAGGGTATTTAAACTCTTTCTT
delStc Saci0074Rev2	TCAACGACATTATTTCTTAAAAAAATTCAAAACGTCAGTTCCCCTAGGTCTATTCGATGT
delStc Sa2156 F	GGTTAGTACTAGAGATTATCATATGGGTAGAACGCAACCGTATTTAAACTCTTTCTTTCA
delStc Sa2156 R	AGTAGAACTCAACTATCTCATCTCTTTGTATTATCCTATCTAAGG TCTATTCGATGTTCT
delSto Sa0554f	CCTTAAATGCTTATACACCAAATACTAAATGTAAATGATAGTGGCAGTGGGTATTTAAAC
delSto Sa0554r	TTAAGCAAAATCCTTAACTCGTTGCAATTAAATGTCGAAGAAATCCCACTGCCTAGGTCT
Saci0074regionF	AAGAGAGGAAGTGGTATTGGC
Saci0074regionR	AACAAGAGGCTCAACAGGC
Saci0554f1	CGCATTTAATTATGCGTATGTGC
Saci0554r1	GCTATTAAAGGAAAGAAGGCAGT
Saci2156regionF	GAACCTTTCTCAGCCCTGT
Saci2156regionR	CGTCTCCCATCTCCTCAAT
SsoCassInt f1	TCAGGTAAGGTTAGTCCATA
SsoCassInt r1	GAGAGTGTAATTTGACTCCT
StoCassIntF1:	GGAAGATCTCCCCTTACTA
StoCassIntR1	TCCTTGATGTTGTTCTTGT
**TLS assays**	
5′ anchor	ACTTACAAGCAATAAATGAGGCAAATGGAACGCCCCCAGTAACAACTCCCAATATCATAT
3′ anchor	(P)ATGTCGACTGCAGAACTAACGACGAATGAAAATATGTCAGGATGGTTGGGGAGTTTCCTT
Downstream scaffold	GCTCAATTTGATATGATATTG
Upstream scaffold	AGTCGACATAGTCAAAGG
Control insert	(P)CAAATTGAGCNCCTTTGACT
oxoG insert	(P)CAAATTGAGCoGCCTTTGACT

*The common sequence for the amplification of the pyrE-lacS dual marker and 5′, 3′, and Tg regions are indicated by capital letters, underlining, bold font, and double lines, respectively.

### Transformation Procedure

The preparation of electrocompetent cells and the transformation protocol were previously described in detail (Suzuki and Kurosawa, 2017). *S. acidocaldarius* (strains DP-1, HM-1, HM-2, and HM-5) electrocompetent cells were cultivated with shaking in XTU medium. Cells at early to midlog phase, in which the optical density of the culture at 600 nm (OD_600_) was ranged from 0.32 to 0.45, were harvested by centrifugation (10,160 × *g* for 15 min at 25°C) using a High Speed Refrigerated Centrifuge Kubota 6500 (KUBOTA), and pellets were washed once in 0.3 volumes of the original culture medium with 20 mM sucrose at room temperature. The OD_600_ was adjusted to 5.9 (2 × 10^9^ cells/mL) on the basis of calculation, and aliquots were frozen at −84°C in an ultralow freezer (Sanyo).

### Construction of DNA Polymerase Deletion Strains

To delete *polB1*, *polB2*, *polB3*, and *dbh*, 1.4 μg of MONSTER-polx was electroporated (15 kV/cm, 9 ms) into 200 μL of DP-1 competent cells in a 2 mm electroporation cuvette (NEPA GENE). Electroporation was performed using a Gene Pulser II system (Bio-Rad). After electroporation, 800 μL of MBS (modified Brock’s basal salt mixture), pH 4.7 (Kurosawa et al., 1998), was added, followed by incubation for 30 min at 77–78°C. The sample was spread onto an XT plate. After seven days of cultivation at 75°C, blue transformant colonies were selected by spraying a 10 mg/mL X-gal solution in 40% DMF diluted with 0.85% sodium chloride solution on the plate, followed by incubation at 75°C for 1 day. The genotype was confirmed using the outer primers (polx-out-F/R) and Emerald Amp MAX PCR Master mix (Takara Bio) under the following conditions: 94°C for 3 min; 30 cycles of 94°C for 30 s, 52 or 56°C for 30 s, and 72°C for 6 min; and a final extension at 72°C for 3 min. Single-colony isolation followed by genotypic analysis using the outer primers was performed at each step for the selection of intermediates and gene deletion strains.

Double- and triple-deletion strains were also constructed. For the construction of the *polB2* and *polB3* double-deletion strain and the *polB2* and *dbh* (*polY*) double-deletion strain, strain HM-1 electrocompetent cells were used. For construction of the *polB3* and *dbh* (*polY*) double-deletion strain, strain HM-2 electrocompetent cells were used. Similarly, for the construction of the *polB2*, *polB3*, and *dbh* triple-deletion strains, strain HM-5 electrocompetent cells were used.

### DNA Polymerase Gene Sequencing Analysis

The undeleted DNAP gene sequences of the deletion strains were checked to confirm whether gene mutations were induced. Undeleted DNAP genes were amplified from cultures of DP-1 and deletion strains using the outer primers (polx-out-F/R) and Emerald Amp MAX PCR Master mix (Takara Bio) under the following conditions: 94°C for 3 min; 30 cycles of 94°C for 30 s, 52°C for 30 s, and 72°C for 6 min; and a final extension at 72°C for 3 min. Each DNAP gene was sequenced using Sanger sequencing technology by the Eurofins Genomics sequencing service.^1^ Sequences were read using the outer primers and inner primers (polx-out-F/R or polx-in-1/2/3) (Table 3). The homology search program was implemented in Genetyx version 12 software.

### Growth After UV Irradiation

As previously described (Suzuki and Kurosawa, 2017), one milliliter of each overnight culture (late-log to stationary phase) of the deletion strains was poured into plastic petri dishes and exposed to UV light (302 nm) using a UV transilluminator (UVM-57; TGK) (20 J/m^2^ per sec) for zero, 20, 40, 60, or 80 s (yielding zero, 400, 800, 1,200, and 1,600 J/m^2^, respectively) at the top of the dish at room temperature. Each irradiated sample was inoculated into 6 mL of XTU liquid medium to yield an initial OD_600_ = 0.005. The cells were cultivated at 75 and 60°C in an air incubator without shaking. Then, the cap of the test tube was closed. Cell growth was monitored thereafter.

### Growth in the Presence of DNA-Damaging Agents

Each overnight culture (late-log to stationary phase) was inoculated into 6 mL of XTU liquid medium containing one type of DNA-damaging agent [cisplatin (Wako) (zero, 20, 30, or 40 μg/mL), 4-nitroquinoline N-oxide (4-NQNO) (TCI) (zero, 0.2, 0.4, 0.6, or 0.8 μg/mL), novobiocin (Nacalai Tesque) (zero, 0.8, 1.2, 2, or 4 μg/mL), or hydroxyurea (Wako) (zero, 0.05, 0.075, or 0.1 mM)] to yield an initial OD_600_ = 0.005. The cells were cultivated at 75 and 60°C in an air incubator without shaking. Then, the cap of the test tube was closed. Cell growth was monitored thereafter.

### Survival Assays

To measure survival of UV-B irradiation, diluted samples (5 μL) of each overnight culture (10^0^–10^–6^ dilution with 20 mM sucrose) were spotted onto XTU plates, and the plates were exposed to UV light (312 nm) using a UV transilluminator (MD-20; WEALTEC) positioned approximately 2.8 cm from the top of the dish at room temperature for zero, 9, 12, or 15 s, respectively. The plates were incubated at 75°C for 8 days and 60°C for 24 days. Survival of UV-C radiation was measured as described previously (Schmidt et al., 1999) by exposing cells suspended in UV-transparent buffer under a germicidal lamp. Samples withdrawn after different exposures were serially diluted and spread-plated on XT under dim red light before incubation.

To measure survival of nitrosoguanidine (MNNG), methyl methanesulfonate (MMS), and mitomycin C (MMC), 200 μL aliquots of the overnight cultures of each strain (late-log to stationary phase) were collected by centrifugation (21,880 × *g* for 1 min at 25°C), and 200 μL of the supernatant was removed. The pellet was suspended in 100 μL of Milli-Q H_2_O, 100 μL of XT liquid medium (pH 3), and 100 μL of Milli-Q H_2_O in the absence or presence of 60 or 100 μg/mL MNNG (SIGMA), 1.5 or 2 mM MMS (Wako), and 60 or 100 μg/mL MMC (Wako) by pipetting and vortexing. After incubation at 75°C for 1 h, the cells were harvested by centrifugation (21,880 × *g* for 1 min at 25°C), washed once in 1 mL of 20 mM sucrose, and suspended in 100 μL of fresh 20 mM sucrose. The diluted samples (5 μL) were prepared as described above and spotted on XTU plates (in duplicate). The plates were incubated at 75°C for 6 days and 60°C for 20 days. For the heat-shock survival test, overnight cultures of each strain (late-log to stationary phase) (50 μL) were heated for 0, 2, 3, or 4 min at 90°C. Then, diluted samples of the cultures were spotted onto plates and incubated.

### Spontaneous Mutation Analysis

The rate of spontaneous mutations that inactivate the *pyrE* gene was determined using the fluctuation technique (Foster, 2006). Approximately 100 independent small liquid cultures (∼200 μl each, in uracil-supplemented media) were grown in microdilution plates until saturation (∼10^8^ cells/ml); the total amount of each culture was plated on medium containing uracil and 5-fluoroorotic acid (FOA), which selects for inactivation of the *pyrE* or *pyrF* gene (Grogan and Gunsalus, 1993). In addition, three or four cultures from each batch were serially diluted and spread on non-selective media in order to count viable cells. The number of FOA-resistant colonies in each culture and the average number of viable cells per culture were used to calculate μ, the average number of mutational events per cell division, using the empirical probability-generating function generated by the *b-z* rates web interface (Gillet-Markowska et al., 2015).

To analyze the mutations in molecular terms, one FOA-resistant colony was randomly picked from each culture and restreaked on media supplemented with uracil. Each restreaked colony was subjected to DNA extraction, PCR of the *pyrE* gene, and chain-termination sequencing. The sequence change and its location in the *pyrE* gene were tabulated to produce mutation spectra. Apparent differences in the frequency of particular types of mutations were evaluated using Fisher’s Exact Test on a 2 × 2 matrix (mutation sub-class vs. all other mutations, strain 1 vs. strain 2) (McDonald, 2014). For these frequency comparisons, the spectrum representing the wild-type background pooled two sets (102 and 61, respectively) of independent FOA-resistant mutants isolated during two fluctuation tests, both using the same conditions as those of this study (Sakofsky et al., 2012; Cong, unpublished).

### Lesion Bypass Assay

Electrocompetent *pyrE*^–^ cells were electroporated with 700 pmol of ssDNA representing 140 nt of the *S. acidocaldarius pyrE* gene transcribed strand, which was produced by ligation of corresponding synthetic oligonucleotides (Table 3, “TLS assays”). Two versions of this ssDNA were used for comparison: a control DNA containing a mixture of four bases at the query position (represented by N in Table 3), and a damaged DNA containing 8-oxoG at the same site (represented by “oG” in Table 3). Ligation of the central segment to the flanking regions was facilitated by short scaffold DNAs complementary to the corresponding two joints (Table 3, “TLS assays”).

The relative efficiency of transformation by the lesion-containing DNA was measured over multiple electroporations and calculated as $\frac{\left(T_{o}/T_{c_{1}}\right)}{\left(T_{t}/T_{c_{2}}\right)}$, where *T*_*o*_, total transformants generated using oxoG oligo; _*T*_*c*1__, total amount of oxoG oligo used; *T*_*t*_, total transformants generated with the control oligo; and *T*_2_, total amount of test oligo used. The identity of the nucleotide inserted opposite the oxoG in individual transformants was determined by restriction analysis. After clonal purification of each transformant, the *pyrE* gene was amplified and treated with restriction endonucleases that discriminate among the four possible bases inserted at the query site (Jain et al., 2020).

## Results

### Deletion of DNA Polymerase Genes

The MONSTER unmarked gene deletion method (Suzuki and Kurosawa, 2017) was applied to the *polB1*, *polB2*, *polB3*, and *dbh* (*polY*) genes of *S. acidocaldarius*. After transformation, 57 colonies/μg MONSTER-polB2, 26 colonies/μg MONSTER-polB3, and 48 colonies/μg MONSTER-Dbh were grown. No colony representing the *polB1* deletion strain could be isolated. After the visualization of blue colonies using X-gal solution, one blue colony was purified via single-colony isolation and analyzed by PCR screening using the outer primers. The blue colonies were intermediate transformants (named HM-1 Int, HM-2 Int, and HM-3 Int, corresponding to PolB2 Int, PolB3 Int, and Dbh Int, respectively). A total of 1.3 × 10^8^ HM-1 Int, 1.2 × 10^8^ HM-2 Int, and 1.5 × 10^8^ HM-3 Int cells were spread on an XTUF plate for pop-out recombination. X-gal visualization revealed 1,269 blue and 60 white colonies of HM-1 Int, 406 blue and 844 white colonies of HM-2 Int, and 1,064 blue and 108 white colonies of HM-3 Int, respectively. Two white colonies were randomly selected for PCR analysis using the outer primers. The genotypes of these colonies exhibited the expected 1.7, 2.3, and 1 kb deletions at the *polB2*, *polB3*, and *dbh* (*polY*) loci, respectively. Thus, *polB2*, *polB3*, and *dbh* deletion strains were constructed and designated *S. acidocaldarius* strains HM-1, HM-2, and HM-3, respectively (Δ*polB2*, Δ*polB3*, and Δ*dbh*, respectively). Similarly, the *polB2*, *polB3*, and *dbh* double-deletion strains were constructed and designated as *S. acidocaldarius* strains HM-4, HM-5, and HM-6 (Δ*polB2*Δ*polB3*, Δ*polB2*Δ*dbh*, and Δ*polB3*Δ*dbh*, respectively). In addition to the double-deletion strains, the construction of the *polB2*, *polB3*, and *dbh* (*polY*) triple-deletion strain was also successful, which was designated as *S. acidocaldarius* strain HM-7 (Δ*polB2*Δ*polB3*Δ*dbh*). We checked all remaining undeleted DNAP gene sequences in knockout strains HM-1–7, revealing that no mutations were introduced into any of the remaining DNAP genes of the knockout strains.

In a parallel set of experiments, prompted by earlier work demonstrating successful replacement of the *polB2* and *polB3* genes individually with selectable cassettes (X-Y Cong, M.S. thesis, University of Cincinnati), the *polB2*, *polB3*, and *dbh* genes (Saci_2156, Saci_0074, and Saci_0554, respectively) were deleted by replacement with heterologous cassettes to generate an otherwise wild-type strain lacking all three DNAPs (see section “Materials and Methods”). Successful deletion of each gene in this strain (RJ11) was confirmed by similar PCR analysis.

### Sensitivity to UV Irradiation

UV irradiation of DNA produces the helix-distorting lesions cyclobutane pyrimidine dimers (CPD) and pyrimidine (6-4) pyrimidine photoproducts (6-4PP) which block DNA polymerization in bacteria and eukaryotes (Courcelle et al., 1999; Lopes et al., 2006; Dorazi et al., 2007). To investigate the relative importance of *S. acidocaldarius* DNAPs in coping with these lesions, we compared the UV sensitivity of single, double, and triple mutants to the corresponding parental strain.

**FIGURE 1:**
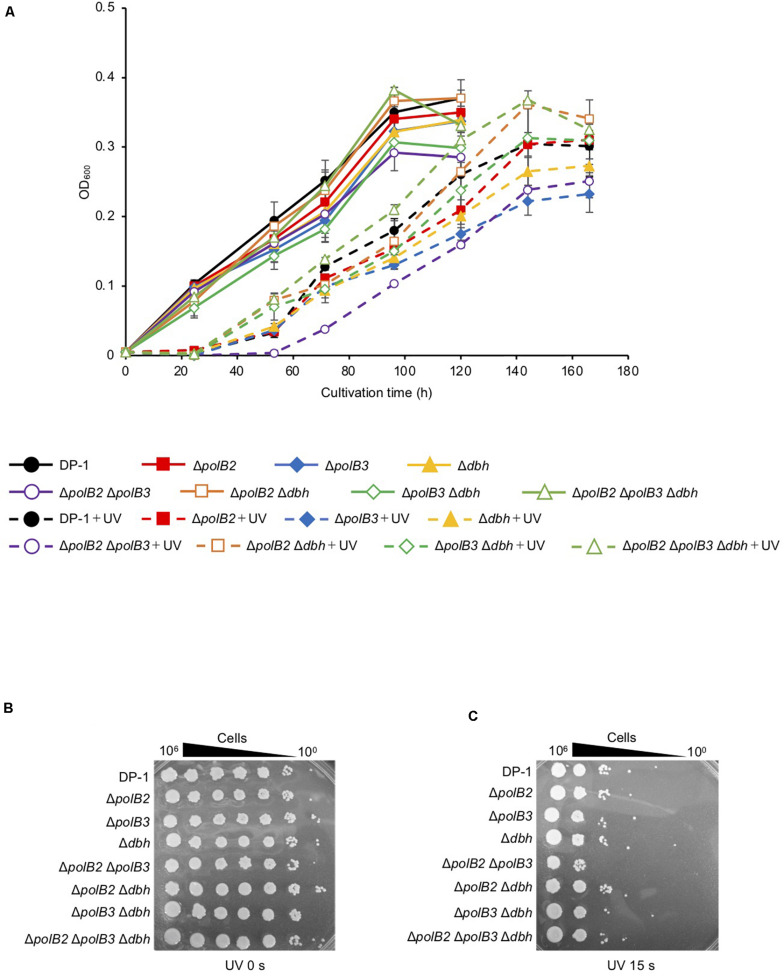
Growth after UV-B irradiation **(A)**. Each overnight culture of the deletion strains was irradiated with UV for 80 s (1600 J/m^2^) and cultivated at 75°C. +UV represents a UV-treated sample. The error bars indicate the mean ± SD calculated using two biological replicates. Closed circle, the growth of DP-1; closed square, the growth of Δ*polB2* (HM-1); closed diamond, the growth of Δ*polB3* (HM-2); closed triangle, the growth of Δ*dbh* (HM-3); open circle, the growth of Δ*polB2*Δ*polB3* (HM-4); opened square, the growth of Δ*polB2*Δ*dbh* (HM-5); open diamond, the growth of Δ*polB3*Δ*dbh* (HM-6); open triangle, the growth of Δ*polB2*Δ*polB3*Δ*dbh* (HM-7). The deletion strains were tested for UV sensitivity **(B,C)**. After UV-B exposure (15 s), diluted samples (10^0^–10^– 6^) of DP-1 and the deletion strains were spotted onto XTU plates and cultivated at 75°C. **(B)** mock-treated samples; **(C)** UV exposure for 15 s.

We characterized the growth properties of deletion strains in liquid medium after UV-B irradiation (zero, 400, 800, 1,200, 1,600 J/m^2^, respectively). No growth retardation of the deletion strains was observed at 75°C after UV irradiation (zero and 800 J/m^2^). However, after UV irradiation (1,200 J/m^2^), the growth of Δ*polB2*Δ*polB3* was slightly retarded compared with that of the parent strain (data not shown). After UV irradiation (1,600 J/m^2^), the difference became more striking, as shown in Figure 1A. The growth curves of the deletion strains and parent strain were nearly the same at 60°C (data not shown). After UV irradiation (400 and 800 J/m^2^), no growth retardation of the deletion strains was observed at 60°C; however, at 1,200 J/m^2^, the growth of Δ*polB2*Δ*polB3* was slightly retarded compared with that of the parent strain (data not shown).

UV survival of deletion strains was measured by plating. Mock-treated and UV-treated samples of the deletion strains and the parent strain were spotted on plates and cultivated at 75°C (Figures 1B,C). The Δ*polB2*Δ*polB3* survival rate was slightly decreased after UV irradiation (9, 12 s) treatment in comparison with that of the parent strain at 75°C; however, negligible sensitivity of other deletion strains to UV irradiation was observed (data not shown). After UV irradiation (15 s), decrease of the Δ*polB2*Δ*polB3* survival rate is more striking (Figure 1C). Experiments also have been performed at 60°C and no difference have been observed (data not shown). The results were therefore consistent with the growth properties of the deletion strains after UV irradiation. Similarly, a different parental strain and Δ*polB2*Δ*polB3*Δ*dbh* were compared with respect to survival as a function of UV-C dose (Table 4). A pronounced “shoulder” was evident up to about 40 J/m^2^ UV-C, but the response of both strains was very similar over the range of doses used (Table 4). Taken together with previous results for polB2 and polB3 deletions (X-Y Cong, M. S. Thesis, University of Cincinnati), the experiments detected sensitivity to UV irradiation primarily for the Δ*polB2*Δ*polB3* strain, as summarized in Table 5.

**TABLE 4:** Survival as a function of UV Dose^1^.

UV dose (J/m^2^)	Log (surviving fraction)^2^	
	Wild-type (RJ12)	Triple-deletion (RJ1101)
0	0	0
10	−0.291 ± 0.086	−0.277 ± 0.021
21	−0.487 ± 0.050	−0.327 ± 0.006
31	−0.579 ± 0.088	−0.485 ± 0.043
42	−0.679 ± 0.011	−0.569 ± 0.041
52	−1.818 ± 0.150	−1.111 ± 0.000

*^1^Results are for UV-C irradiation of cell suspensions assayed by colony formation (two independent experiments).*

*^2^Values are average ± one-half of the range.*

**TABLE 5:** Sensitivity of deletion strains to various types of damage.

Treatment	Δ*polB2*		Δ*polB3*		Δ*dbh*		Δ*polB2* Δ*polB3*		Δ*polB2* Δ*dbh*		Δ*polB3*Δ*dbh*		Δ*polB2* Δ*polB3* Δ*dbh*	
	75°C	60°C	75°C	60°C	75°C	60°C	75°C	60°C	75°C	60°C	75°C	60°C	75°C	60°C
UV	−	−	−	−	−	−	+	+	−	−	−	−	−	−
cisplatin	−	−	+	+	+	−	+	++	+	+	++	+	++	++
4-NQNO	±	+	±	+	±	+	++	++	±	+	±	++	±	++
MMC	−	−	−	−	−	−	++	++	−	−	−	−	−	−
MNNG	−	−	−	−	−	−	++	++	−	−	+	+	+	+
Heat-shock	+	+	+	+	+	+	++	++	±	±	+	+	+	+
Novobiocin	−	−	−	±	−	±	−	++	−	±	−	++	−	++
HU	++	−	−	−	+	−	±	+	++	++	++	++	++	++

*The sensitivity of deletion strains to various types of DNA damage is summarized. −, ±, +, and ++ indicates no sensitivity, marginal sensitivity, sensitivity, and significant sensitivity, respectively. All the mutants showed no sensitivity to MMS.*

### Sensitivity to Chemical Mutagens

**FIGURE 2:**
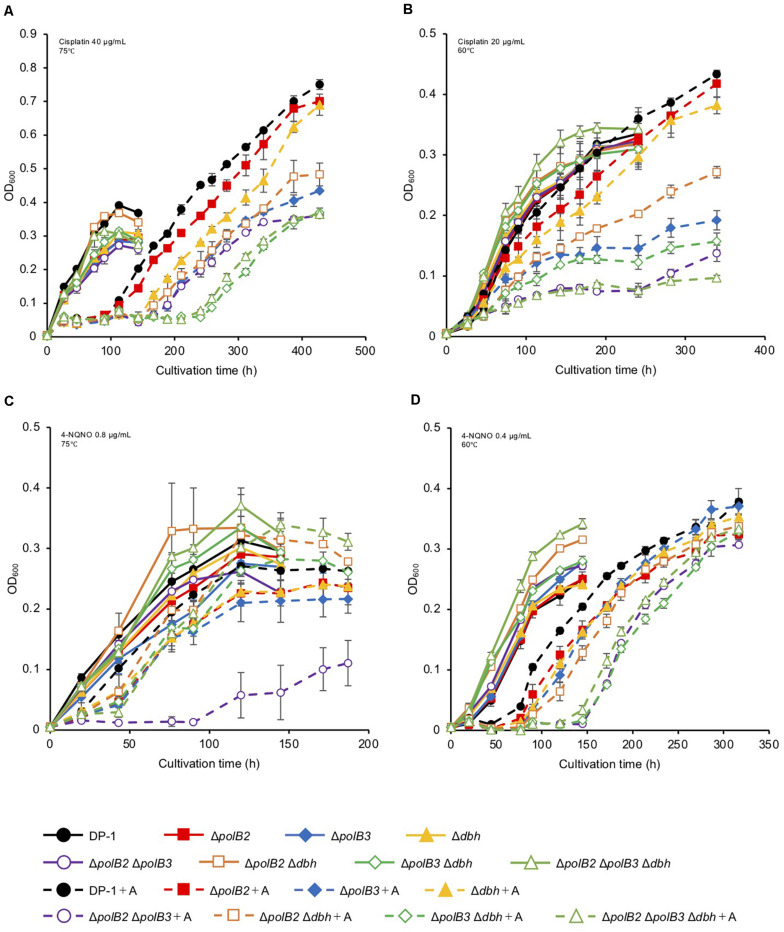
Growth in the presence of DNA-damaging agents. Overnight cultures of each deletion strain and DP-1 were inoculated into liquid medium in the presence of DNA-damaging agents {cisplatin [40 **(A)** and 20 μg/mL **(B)**] and 4-NQNO [0.8 **(C)** and 0.4 μg/mL **(D)**]} and cultivated at 75 and 60°C, respectively. +A represents the growth curve in the presence of DNA-damaging agents. The error bars indicate the mean ± SD, calculated using two biological replicates. Closed circle, the growth of DP-1; closed square, the growth of Δ*polB2* (HM-1); closed diamond, the growth of Δ*polB3* (HM-2); closed triangle, the growth of Δ*dbh* (HM-3); open circle, the growth of Δ*polB2*Δ*polB3* (HM-4); open square, the growth of Δ*polB2*Δ*dbh* (HM-5); open diamond, the growth of Δ*polB3*Δ*dbh* (HM-6); open triangle, the growth of Δ*polB2*Δ*polB3*Δ*dbh* (HM-7).

To test for possible effects of other DNA lesions, we investigated the sensitivity of the deletion strains to certain chemicals known to generate DNA lesions. Most experiments evaluated multiple concentrations of these chemicals at two incubation temperatures (75 or 60°C). Cisplatin and 4-NQNO, for example, are known to produce intra-strand cross-links and bulky adducts, respectively. Deletion and parental strains were incubated in growth medium with or without cisplatin (20–40 μg/mL). In the presence of cisplatin (30 μg/mL), the growth of Δ*polB2*Δ*dbh* and Δ*polB2*Δ*polB3*Δ*dbh* was slightly delayed compared with that of the parent strain at 75°C (data not shown). In the presence of cisplatin (40 μg/mL), the growth of all the deletion strains except for Δ*polB2* was delayed compared with that of the parent strain (Figure 2A). Notably, the growth of Δ*polB3*Δ*dbh* and Δ*polB2*Δ*polB3*Δ*dbh* was retarded compared with that of Δ*polB3*, Δ*dbh*, Δ*polB2* Δ*polB3*, and Δ*polB2* Δ*dbh* (Figure 2A). At 60°C, in the presence of cisplatin (20 μg/mL), the growth of all deletion strains except for Δ*polB2* and Δ*dbh*, was retarded compared with that of the parent strain (Figure 2B). Specifically, the growth of Δ*polB2*Δ*polB3* and Δ*polB2*Δ*polB3*Δ*dbh* was retarded compared to that of Δ*polB3*, Δ*polB2* Δ*dbh*, and Δ*polB3* Δ*dbh* (Figure 2B). These results suggested that deleting *polB2* alone did not increase sensitivity to cisplatin whereas deleting *polB3* and *dbh* or *polB2 polB3* and *dbh* increased cisplatin sensitivity at 75°C. At 60°C, Δ*polB2* and Δ*dbh* did not appear to be sensitive to cisplatin, but Δ*polB2*Δ*polB3* and Δ*polB2*Δ*polB3*Δ*dbh* exhibited significant sensitivity (Table 5).

The growth properties of the deletion strains was examined in the presence or absence of 4-NQNO (0.2–0.8 μg/mL) at 75 or 60°C. At 75°C, in the presence of 0.6 μg/mL 4-NQNO, the growth of Δ*polB2*Δ*polB3* was slightly retarded compared with that of the parent strain (data not shown). At 0.8 μg/mL, the difference became more striking (Figure 2C). Similarly, the growth properties of the deletion strains were examined at 60°C (Figure 2D). In the presence of 4-NQNO (0.2 μg/mL), the growth of all deletion strains was slightly retarded compared with that of the parent strain (data not shown). The growth of Δ*polB2*Δ*polB3*, Δ*polB3*Δ*dbh*, and Δ*polB2*Δ*polB3*Δ*dbh* was more delayed in the presence of 0.4 μg/mL 4-NQNO (Figure 2D) than 0.2 μg/mL 4-NQNO (data not shown). Thus, the Δ*polB2*Δ*polB3* strain was sensitive to 4-NQNO at both 75 and 60°C, whereas the Δ*polB3*Δ*dbh* and Δ*polB2*Δ*polB3*Δ*dbh* strains exhibited significant sensitivity only at 60°C (Table 4).

**FIGURE 3:**
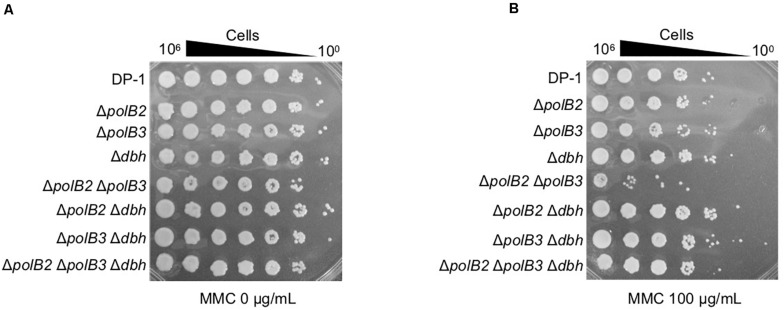
MMC sensitivity. After MMC treatment (10 mM), diluted samples (10^0^–10^– 6^) of DP-1 and the deletion strains were spotted onto XTU plates and cultivated at 75°C. **(A)** mock-treated samples; **(B)** MMC-treated samples.

To analyze the sensitivity of the deletion strains to MMC, which induces interstrand DNA crosslinks, mock- and MMC-treated (zero, 60, and 100 μg/mL) aliquots of the deletion strains and the parent strain were spotted on plates. Δ*polB2*Δ*polB3* survival after MMC (60 μg/mL) treatment was decreased in comparison with that of the parent strain at 75°C (data not shown), a difference that became more striking under MMC (100 μg/mL) treatment (Figure 3). The results of the experiments performed at 60°C were same as those at 75°C. Interestingly, no sensitivity of Δ*polB2*Δ*polB3*Δ*dbh* to MMC was observed (Figure 3B). These results indicated that Δ*polB2* Δ*polB3* was significantly sensitive to MMC (Table 5).

**FIGURE 4:**
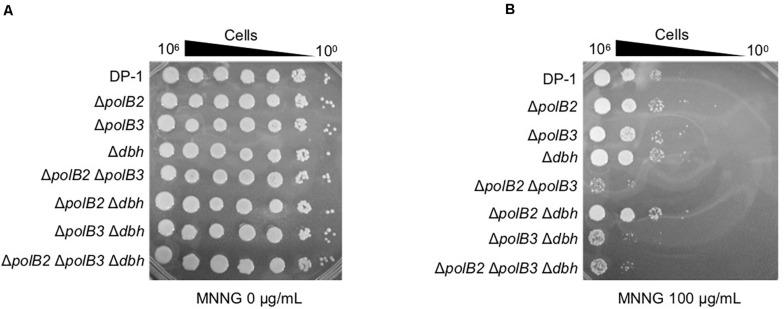
MNNG sensitivity. After MNNG treatment (100 μg/mL), diluted samples (10^0^–10^– 6^) of DP-1 and the deletion strains were spotted onto XTU plates and cultivated at 75°C. **(A)** mock-treated samples; **(B)** MNNG-treated samples.

In order to investigate additional forms of DNA damage, we treated cells with MNNG, which forms the highly mutagenic *O*^6^-methylguanine, and MMS, which forms 7-methylguanine and 3-methyladenine (Lindahl, 1993). Mock-treated and MNNG- or MMS-treated samples of the deletion strains and parent strain were spotted on plates and cultivated at 75 and 60°C. The survival of Δ*polB2*Δ*polB3*, Δ*polB3*Δ*dbh*, and Δ*polB2*Δ*polB3 Δdbh* strains after treatment with MNNG (60 μg/mL) was decreased in comparison with that of the parent strain at 75°C (data not shown), and this difference became more striking at 100 μg/mL (Figure 4). The results of the experiments performed at 60°C were same as those at 75°C. In contrast, the deletion strains did not show sensitivity to MMS at 75 and 60°C (data not shown). Under these conditions, therefore the Δ*polB2*Δ*polB3* was obviously sensitive to MNNG, while the Δ*polB3*Δ*dbh* and Δ*polB2*Δ*polB3*Δ*dbh* strains were less sensitive. None of the deletion strains showed increased sensitivity to MMS, however.

### Sensitivity to Heat-Shock Treatment

**FIGURE 5:**
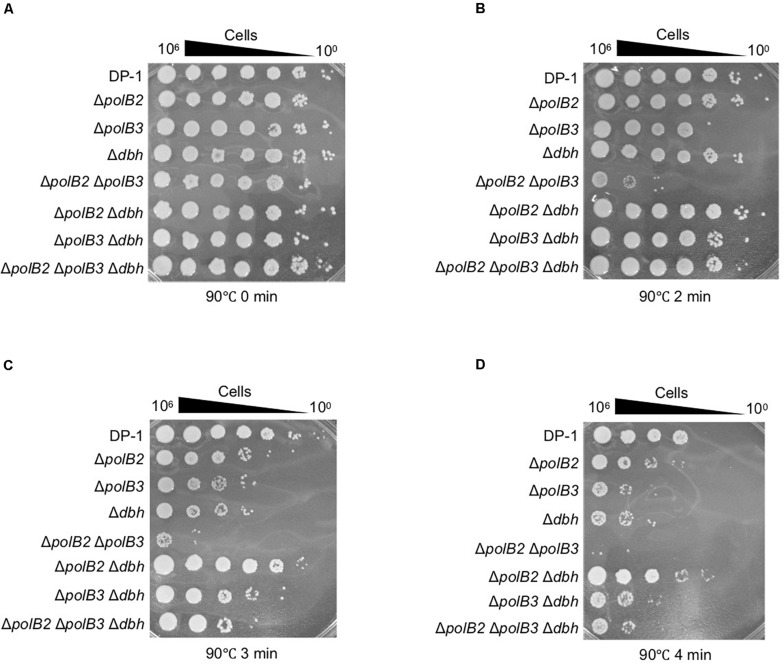
Heat-shock sensitivity. After treatment at 90°C (0, 2, 3, and 4 min), diluted samples (10^– 6^–10^0^) of DP-1 and the deletion strains were spotted onto XTU plates and cultivated at 75°C. **(A)** mock-treated samples; **(B–D)** heat-shock for 2, 3, and 4 min at 90°C, respectively.

To examine the sensitivity of deletion strains to heat-shock, aliquots of the deletion strains and parent strain that had been mock treated or heat-treated at 90°C (2, 3, and 4 min) were spotted onto plates and cultivated at 75 or 60°C for colony formation. Survival of Δ*polB2*Δ*polB3* after 2 min at 90°C was dramatically less that of the parent strain at 75°C (Figures 5A,B). Longer heating (3 or 4 min) revealed sensitivity of the Δ*polB2*, Δ*polB3*, Δ*dbh*, Δ*polB3* Δ*dbh*, Δ*polB2*Δ*polB3*, and Δ*polB2* Δ*polB3* Δ*dbh* strains (Figures 5C,D). For these various strains and treatments here were no apparent differences in survival for 75 vs. 60°C plating. These results indicated that Δ*polB2*, Δ*polB3*, Δ*dbh*, Δ*polB3* Δ*dbh*, and Δ*polB2* Δ*polB3* Δ*dbh* were sensitive to heat stress and that Δ*polB2*Δ*polB3* exhibited greater sensitivity than these constructs (Table 5).

### Sensitivity to DNA Replication Inhibitors

**FIGURE 6:**
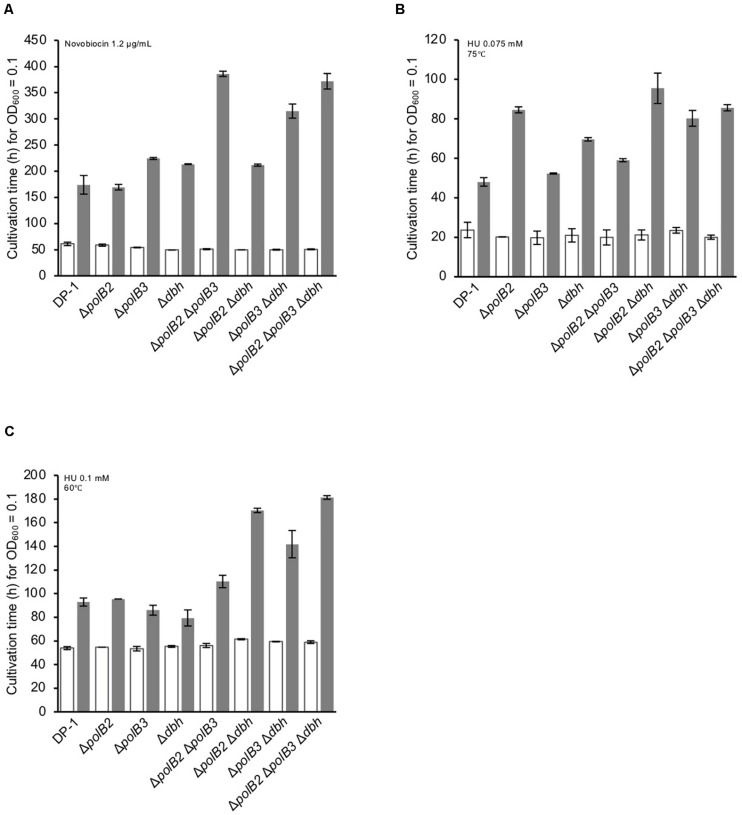
Cultivation time to reach OD_600_ = 0.1. Overnight cultures of the deletion strains and DP-1 were inoculated into liquid medium in the presence of a DNA replication inhibitor {novobiocin (1.2 μg/mL) **(A)** and HU [0.075 **(B)** and 0.1 mM **(C)**]} and cultivated at 75 and 60°C. The cultivation time was calculated from the growth curves of the deletion strains in the presence of a DNA replication inhibitor. White and gray bars indicate cultivation in the absence and presence of the DNA replication inhibitor, respectively. The error bars indicate the mean ± SD, calculated using two biological replicates.

The growth properties of the deletion strains were examined in the presence or absence of novobiocin, which has been observed to inhibit DNA replication in *S. acidocaldarius* (Hjort and Bernander, 2001). At 60°C, novobiocin (0.8 μg/mL), retarded the growth of Δ*polB3* and Δ*dbh*, but had a limited effect on Δ*polB2* (data not shown). The growth of Δ*polB2* Δ*dbh* was nearly the same as that of Δ*polB3* and Δ*dbh*. These results suggested that deletion of *polB2* did not measurably increase novobiocin sensitivity at 60°C, whereas deleting *polB3*, *dbh*, or *polB2* and *dbh* increased it somewhat, and deleting *polB2* and *polB3*, *polB3* and *dbh*, or all three genes increased it more. At 1.2 μg/mL, these differences became more striking (Figure 6A), as summarized in Table 5. At 75°C, no growth retardation of the deletion strains was observed compared with that of the parent strain in the presence of novobiocin (2 and 4 μg/mL) (data not shown).

HU inhibits many ribonucleotide reductases and is widely used as a general inhibitor of DNA synthesis, although it appears to perturb chromosome replication in *S. solfataricus* by an unknown mechanism (Liew et al., 2016). The growth properties of the deletion strains were examined in the presence or absence of HU (0.05, 0.075, and 0.1 mM) at 75 and 60°C. In the presence of HU (0.05 mM), the growth of Δ*polB2*Δ*dbh* was slightly delayed compared with that of the parent strain at 75°C (data not shown). In addition, in the presence of HU (0.075 mM), the growth of all of the deletion strains except for Δ*polB3* was delayed compared with that of the parent strain (Figure 6B). The growth of Δ*polB2* Δ*polB3* was marginally retarded in comparison with that of the parent strain. Notably, the growth of Δ*polB2*, Δ*polB2*Δ*dbh*, Δ*polB3*Δ*dbh*, and Δ*polB2*Δ*polB3*Δ*dbh* was significantly retarded compared with that of Δ*dbh* and Δ*polB2* Δ*polB3*. However, in the presence of HU (0.1 mM), the growth curve of the deletion strains was nearly the same as that of the parent strain (data not shown). At 60°C, no growth retardation of the deletion strains was observed in the presence of HU (0.05 mM) (data not shown). However, in the presence of HU (0.075 mM), the growth of Δ*polB2*Δ*dbh* was slightly delayed compared with that of the parent strain (data not shown). In addition, in the presence of HU (0.1 mM), in contrast to the single knockout strains, the growth of the double- and triple-deletion strains was delayed compared with that of the parent strain (Figure 6C). In particular, the growth of Δ*polB2*Δ*dbh*, Δ*polB3*Δ*dbh*, and Δ*polB2*Δ*polB3*Δ*dbh* was retarded compared with that of the parent strain. These results indicated that Δ*polB2* Δ*polB3* and Δ*polB3* Δ*dbh* were slightly sensitive and sensitive to HU, respectively, and that Δ*polB2* Δ*dbh* and Δ*polB2* Δ*polB3* Δ*dbh* exhibited significant susceptibility at 60°C (Table 5).

### Spontaneous Mutation

To allow for the possibility that some of the non-essential DNA enzymes of *S. acidocaldarius* may be able to substitute for each other under laboratory conditions, we focused our analysis of spontaneous mutation on comparing the triple-polymerase mutant RJ1101 (which has no other genes deleted) with the corresponding Pol^+^ strain RJ12 and *dbh* strain CS2 (Sakofsky et al., 2012). Fluctuation assays revealed no difference in the overall rate of forward mutation of this target gene under these conditions: 2.8 × 10^–7^ for RJ1101 vs. 2.9 × 10^–7^ for RJ12 (95% confidence interval 2.42–3.35 × 10^–7^ vs. 2.20–3.64 × 10^–7^, respectively). We note that inactivation of *dbh* (*polY*) by itself also had a limited effect on the overall forward mutation rate in *S. acidocaldarius*, despite its well-defined impact on a particular class of spontaneous mutation (Sakofsky et al., 2012).

Therefore, to investigate effects on particular classes of mutation, we determined the spontaneous mutation spectrum of the triple-deletion strain by sequencing the *pyrE* gene of independent mutants. Nearly all (>95%) of the Foa^*R*^ mutants of strain RJ1101 revealed a single mutation in the *pyrE* gene. These mutations and their positions were then compiled to yield a set of 94 independent events, which we compared to corresponding sets of independent *pyrE* mutations in wild-type and *dbh*^–^ (*polY*^–^) strains (Sakofsky et al., 2012). The resulting three sets of independent mutations are summarized in Table 6.

**TABLE 6:** Spontaneous *pyrE* mutations^1^.

	Wild-type^2^	Fraction	RJ1101	Fraction	CS2^3^	Fraction
**Total**	163	1.000	94	1.000	110	1.000
**Primary types^4^**						
A-tract +	22	0.135	12	0.128	5	0.045
A-tract −	8	0.049	7	0.074	8	0.073
C-tract +	4	0.025	2	0.021	3	0.027
C-tract −	3	0.018	2	0.021	3	0.027
G-tract +	27	0.166	14	0.149	18	0.164
G-tract −	28	0.172	6	0.064	7	0.064
T-tract +	8	0.049	0	0.000	2	0.018
T-tract −	6	0.037	2	0.021	0	0.000
+A	2	0.012	0	0.000	1	0.009
−A	1	0.006	0	0.000	0	0.000
+C	0	0.000	0	0.000	0	0.000
−C	1	0.006	0	0.000	1	0.009
+G	1	0.006	0	0.000	1	0.009
−G	0	0.000	0	0.000	0	0.000
+T	0	0.000	1	0.011	2	0.018
−T	0	0.000	0	0.000	0	0.000
A to C	1	0.006	0	0.000	0	0.000
A to G	1	0.006	0	0.000	2	0.018
A to T	2	0.012	1	0.011	0	0.000
C to A	3	0.018	8	0.085	6	0.055
C to G	1	0.006	0	0.000	1	0.009
C to T	0	0.000	4	0.043	1	0.009
G to A	9	0.055	3	0.032	14	0.127
G to C	1	0.006	0	0.000	1	0.009
G to T	7	0.043	18	0.191	21	0.191
T to A	2	0.012	0	0.000	1	0.009
T to C	6	0.037	2	0.021	0	0.000
T to G	2	0.012	0	0.000	1	0.009
indel < 6	1	0.006	4	0.043	1	0.009
dupl > 5	13	0.080	4	0.043	9	0.082
del > 5	3	0.018	4	0.043	1	0.009
Complex	0	0.000	0	0.000	0	0.000
**Secondary types^5^**						
A tracts	30	0.186	19	0.202	13	0.118
C tracts	7	0.043	4	0.043	6	0.055
G tracts	55	0.342	20	0.213	25	0.227
T tracts	14	0.087	2	0.021	2	0.018
A:T tract +	30	0.184	12	0.128	7	0.064
G:C tract +	31	0.190	16	0.170	21	0.191
Tracts +	61	0.379	28	0.298	28	0.255
Tracts −	45	0.280	15	0.160	18	0.164
fs in tracts	106	0.658	42	0.447	46	0.418
Single nt +	3	0.019	1	0.011	4	0.036
Single nt −	2	0.012	0	0.000	1	0.009
Isolated fs	5	0.031	1	0.011	5	0.045
Total fs	111	0.689	43	0.457	51	0.464
A:T to G:C	7	0.043	2	0.021	2	0.018
A:T to T:A	4	0.025	1	0.011	1	0.009
G:C to C:G	2	0.012	0	0.000	2	0.018
G:C to A:T	9	0.056	7	0.074	15	0.136
G:C to T:A	10	0.062	27	0.287	27	0.245
BPS at A	4	0.025	1	0.011	2	0.018
BPS at C	4	0.025	12	0.128	8	0.073
BPS at G	17	0.106	22	0.234	36	0.327
BPS at T	10	0.062	2	0.021	2	0.018
BPS at G:C	21	0.130	34	0.362	44	0.400
BPS at A:T	14	0.087	3	0.032	4	0.036
Transversions	19	0.118	28	0.298	31	0.282
Transitions	16	0.099	9	0.096	17	0.155
Total BPS	35	0.217	37	0.394	48	0.436
Large indels	16	0.099	14	0.149	11	0.100

*^1^All spectra exclude frameshifts in the 7A tract at nt 545.*

*^2^ATCC 33909 (laboratory designation DG185); represents mutants pooled from two experiments (Sakofsky et al., 2012; Cong, unpublished).*

*^3^See Sakofsky et al. (2012).*

*^4^+, indicated nt added; −, indicated nt removed; dupl, tandem duplication; indel, insertion/duplication or deletion.*

*^5^Combinations of primary mutation types. fs, frameshift; BPS, base-pair substitution.*

Comparing three broad mechanistic categories suggested that the three strains did not differ significantly with respect to the frequency of large events, i.e., deletions and tandem duplications. The relative abundance of frameshifts vs. BPS events was affected, however, being markedly lower in the spectra of the two *dbh* mutant strains than in wild-type (Table 7).

**TABLE 7:** Statistical evaluation of mutation spectra^1^.

Mutation type^2^	Strains compared^3^		
	Wild-type vs. RJ1101	Wild-type vs. CS2	RJ1101 vs. CS2
A-tract +	1	0.021	0.043^4^
G-tract −	0.013	0.009	1
C to A	0.02	0.164	0.418
C to T	0.018	0.403	0.183
G to A	0.544	0.046	0.020^4^
G to T	0.00027	0.00015	1
A:T tract +	0.294	0.0039	0.148
fs in tracts	0.0017	0.003	0.777
Total fs	0.00057	0.00042	1
GC: to A:T	0.596	0.028	0.179
BPS at C	0.002	0.074	0.239
BPS at G	0.0067	8.4E-06	0.162
Total BPS	0.00252	0.00027	0.571

*^1^P-values calculated by Fisher’s exact test for the indicated class of mutation vs. all other classes for the two strains indicated (see section “Materials and Methods”).*

*^2^+, tract expanded by one bp; −, tract shortened by one bp; fs, frameshift; BPS, base-pair substitution.*

*^3^Strain CS2 is *dbh*^–^, strain RJ1101 is Δ*polB2* Δ*polB3*Δ*dbh* (*polY*) (Table 2).*

*^4^Values that distinguish the triple mutant from wild-type.*

Less than half of the frameshift sub-classes were affected by DNAP genotype, and most of the effects reflected *dbh* (*polY*) status (Table 7). Only one frameshift sub-class discriminated between triple-deletion strain and *dbh* single mutant; this was the expansion of A tracts (Table 6), which was elevated in the triple mutant relative to both *dbh* and wild-type and yielded a *P*-value less than 0.05 (Table 7). Similarly, several sub-classes of base-pair substitutions indicated apparent differences among the three strains, but, as in the case of frameshifts, most of these correlated with *dbh* (*polY*) inactivation. The most notable exception was G to A transitions, which were elevated in the *dbh* single mutant relative to wild-type or the triple-deletion strain, and yielded a *P*-value below 0.05 (Table 7).

Thus, detailed comparisons of distinct sub-classes of mutations in the three *pyrE* spectra indicated only the possibility that deleting *polB2* and *polB3* in a *dbh* (*polY*) background may (i) encourage the expansion of A:T tracts oriented with A in the top strand and (ii) avoid transition mutations at G:C bp oriented with G in the top strand. Both of these sub-classes represent strand-specific events. *A priori*, strand specificity does not have an obvious mechanistic interpretation in this context, aside from possible effects of the direction of replication or transcription. We note, however, that in both cases the strand orientation meeting the statistical criterion is the one that is most common within the mutational target.

### Bypass of a DNA Lesion

In order to analyze TLS past a specific lesion *in vivo*, we used a genetic assay to score individual oxoG bypass events in the chromosomes of normal, Δ*dbh*, and triple-deletion polymerase mutants of *S. acidocaldarius*. The assay selects for cells that have been genetically transformed by synthetic single-stranded oligonucleotides carrying 8oxoG at a synonymous position within the *pyrE* gene sequence (Jain et al., 2020). Thus, successful transformation requires incorporation of the oligonucleotide into the recipient chromosome and bypass of the lesion in the first round of replication, but any of the four nucleotides inserted opposite the lesion generates a transformant. At this position each nucleotide creates a distinct restriction site, however, allowing the inserted nucleotide to be identified by restriction assays of the PCR products (Jain et al., 2020).

We compared *pol*^+^, Δ*dbh*, and Δ*pol2*Δ*pol3*Δ*dbh* strains with respect to transformation by oxoG-containing DNA and the specificity of oxoG bypass. As expected, the *pol*^+^ recipient demonstrated the highest relative transformation efficiency (0.659), representing a limited impact of G oxidation on oligonucleotide incorporation and replication in normal cells. The corresponding transformation efficiency of the Δ*dbh* recipient strain was about 10% of this value (0.068), indicating that loss of the Y-family DNAP Dbh compromised the cell’s ability to tolerate oxidative damage, consistent with the biochemical and genetic properties of this DNAP (Sakofsky et al., 2012; Sakofsky and Grogan, 2015; Jain et al., 2020). The triple polymerase mutant yielded a relative transformation efficiency similar to that of the *Δdbh* recipient. Thus, additional loss of PolB2 and PolB3 did not significantly affect the overall success rate of incorporating and bypassing a specific oxoG. Analysis of the triple deletion mutant detected only insertion of A opposite oxoG, similar to the results obtained from corresponding transformation of the *Δdbh* recipient (Table 8).

**TABLE 8:** Bypass of oxoG *in vivo*^1^.

	Control DNA		oxoG DNA			Nucleotide inserted		
Recipient	Total μmol	Total trans-formants	Total μmol	Total trans-formants	Relative efficiency	Trans-formants scored	C	A
Wild-type	5.38	162	52.45	42	0.659	27	27	0
Δ*dbh*	23.04	129	142.08	23	0.068	19	1	18
Δ*pol2*Δ*pol3*Δ*dbh*	21.5	181	144.38	27	0.088	25	0	25

*^1^Indicated strains were transformed by undamaged or oxoG-containing oligonucleotides (see section “Materials and Methods”). Data are compiled from multiple experiments, each involving multiple electroporations.*

## Discussion

To experimentally define the roles and interactions of four DNAPs in DNA replication and repair in crenarchaea, we attempted to delete the *polB1*, *polB2*, *polB3*, and *dbh* (*polY*) genes in *S. acidocaldarius* singly and in combination. The results demonstrated that it was possible to construct various Δ*polB2*, Δ*polB3*, and Δ*dbh* strains, including triple-deletion strains (Δ*polB2* Δ*polB3* Δ*dbh*), but not a Δ*polB1* strain. These results provide evidence that PolB1 is the replicative DNAP in *S. acidocaldarius* and allowed us to evaluate functional contributions of PolB2, PolB3, and Dbh to genome replication and repair.

Confirming the functional roles of DNAPs in archaea provides a valuable perspective regarding the evolution of DNA replication systems in cellular organisms. Bacteria and eukarya differ with respect to the DNAP used for genome replication (C-family vs. B-family, respectively) and functional specialization within the replisome (illustrated by eukaryotes’ use of Pol∂ for lagging-strand synthesis and Polε for leading-strand synthesis) (Kunkel and Burgers, 2008; Sarmiento et al., 2014). Crenarchaea generally encode only B-family polymerases, implying that they employ at least one of these for genome replication. Several euryachaea, however, have both B- and D-family polymerases. In one case (*Halobacterium*), both polymerases appear to be essential (Berquist et al., 2007), whereas in another (*Thermococcus*) only the D-family enzyme is essential (Cubonová et al., 2013; Kushida et al., 2019). The latter situation, which also has been reported for *Methanococcus maripaludis* (Sarmiento et al., 2013), implies that the single D-family polymerase replicates both leading and lagging arms of replication forks in these euryarchaea.

The B-family polymerases form sub-families, and the broad conservation of the PolB3 sub-family has been suggested as evidence that these polymerases may replicate the genomes of crenarchaea (Makarova and Koonin, 2013; Makarova et al., 2014). On the other hand, Lundgren and Bernander (2007) suggested that the PolB1 of *S. acidocaldarius* performs both leading- and lagging-strand syntheses based on the temporal patterns of transcription of all four polymerases with respect to the cell-division cycle. More recently, biochemical properties of the B-family polymerases of *Sulfolobus* and related genera have been investigated, and argue that the PolB1 enzymes play this replicative role (Choi et al., 2011; Bauer et al., 2012; Peng et al., 2016; Yan et al., 2017), and this conclusion is supported by functional genetic criteria in *S. acidocaldarius* (this study) and closely related crenarchaea (Feng et al., 2020). The recent evidence that *Sulfodiicoccus acidiphilus*, a member of the order *Sulfolobales* lacks PolB3 (Sakai and Kurosawa, 2019) also strongly supports a replicative role of PolB1.

In the present study, *polB3* deletion strains of *S. acidocaldarius* were readily constructed, whereas no *polB1* deletion strain could be isolated under the same conditions. In addition, it was possible to isolate triple*-*deletion strains lacking PolB2, PolB3, and Dbh (PolY), and the growth or survival of the triple-deletion mutants were nearly the same as that of the parent strain under some of the conditions we tested. These results indicate that *S. acidocaldarius* PolB1 is sufficient for supporting normal growth and also a significant level of repair or tolerance of DNA damage by various agents, including UV. The results accordingly argue that PolB1 is the main replicative DNAP and is solely responsible for the replication of both strands of the genome in Crenarchaea, and that the broad conservation of PolB3 in the TACK superphylum reflects a cellular function that is generally not essential for cellular viability.

### Polymerase Involvement in Repair or Tolerance of DNA Damage

In addition to defining the functional importance of PolB1, successful construction of multiple DNAP deletion strains also allowed experimental analysis of the non-essential DNAPs and their functional interaction. Given the fact that accessory DNAPs often affect cellular survival of DNA damage or the accuracy of DNA replication, the present study, investigated the impact of polymerase removal on spontaneous mutation and survival of various stress conditions. Deleting combinations of non-essential DNAPs resulted in various levels of sensitivity to certain DNA-damaging treatments. The patterns of sensitivity, compared with the genotypes of the corresponding strains, provide a genetic assessment of functional roles of non-essential DNAPs for replication and repair of *Sulfolobus* genomes.

The mechanisms by which archaea cope with helix-distorting DNA lesions (including UV photoproducts, intrastrand crosslinks, and bulky adducts) remain a significant question for archaeal molecular biology (White, 2007; Grogan, 2015; White and Allers, 2018; Suzuki and Kurosawa, 2019b). Hyperthermophilic archaea, including *Sulfolobales*, encode homologues of eukaryotic NER proteins (XPF, XPG, XPB, and XPD) (Rouillon and White, 2011; Grogan, 2015; White and Allers, 2018), yet genetic analysis has demonstrated that, of these proteins, only the XPF/Hef endonuclease (Hef is euryarchaeal XPF) contributes significantly to survival of DNA damage (Fujikane et al., 2010; Zhang et al., 2013; Suzuki and Kurosawa, 2019a).

With respect to the functional roles of archaeal DNAPs, our results indicated that the Δ*polB2*Δ*polB3* combination made *S. acidocaldarius* sensitive to UV (Figure 1 and Table 5). The limited magnitude of this effect and its apparent absence in single mutants suggest that PolB2 and PolB3 make contributions to survival of UV photoproducts in which each can substitute to a large extent for the other. A contribution of TLS in the survival of bulky lesions has been suggested by *in vitro* studies in which *S. solfataricus* Dpo4 bypassed benzyl adducts, whereas the PolB2 and PolB3 enzymes of *S. solfataricus* did not (Choi et al., 2011; see Table 1), and in which Dpo4 replicated past cisplatin lesions (Boudsocq et al., 2001). The PolB2 polymerases are distinct from the other DNAPs of *Sulfolobales* in being induced by UV (Fröls et al., 2007; Götz et al., 2007; Feng et al., 2020). In addition, phenotypic analyses indicate that the PolB2 of a strain related to *S. solfataricus* increases survival of UV-C, cisplatin, and NQNO (Feng et al., 2020). In the present study, Δ*polB3* and Δ*dbh* deletion strains of *S. acidocaldarius* were somewhat sensitive to cisplatin at 75°C and the Δ*polB3*Δ*dbh* double-deletion strain was more sensitive than both single mutants (Figure 2A). This provides genetic evidence that both PolB3 and Dbh contribute to the cellular survival of cisplatin-induced damage, similar to a previous report investigating Dpo4 in *S. solfataricus* (Wong et al., 2010). We also found that PolB2, PolB3, and Dbh were important for surviving 4-NQNO treatment, although the effect was greater at low temperature (60°C), and thus may reflect a combination of multiple stresses on chromosomal replication.

Although the observed sensitivity (Table 5) varied considerably among treatments, relative sensitivity of the Δ*polB2*Δ*polB3* double mutant compared to the other genotypes represents a recurring pattern. Similar to the UV and 4-NQNO results, the Δ*polB2*Δ*polB3* strain exhibited the highest sensitivity to MMC, whereas the corresponding single-deletion strains were not sensitive (Figure 3). The alkylating agent MNNG revealed a related pattern, in that only the Δ*polB2*Δ*polB3*, Δ*polB3*Δ*dbh*, and Δ*polB2*Δ*polB3*Δ*dbh* strains exhibited sensitivity (Figure 4 and Table 5). Similarly, Δ*polB2* Δ*polB3*, Δ*polB3*Δ*dbh*, and Δ*polB2* Δ*polB3*Δ*dbh* were sensitive to novobiocin to the same degree, and the other genotypes were not as sensitive. HU revealed a similar pattern, in that double-deletion strains were sensitive to HU at 60°C, but the *polB2*, *polB3*, and *dbh* single-deletion strains were not sensitive (Figure 6C and Table 5). Specifically, Δ*polB2* Δ*dbh* exhibited the strongest sensitivity to HU, followed by Δ*polB3* Δ*dbh* and Δ*polB2* Δ*polB3*. In addition, Δ*polB2* Δ*dbh* and Δ*polB2* Δ*polB3*Δ*dbh* were sensitive to HU to the same degree. Finally, increased sensitivity to the relatively non-specific stress represented by heat-shock, was increased by deleting any of the DNAP genes individually, but was greatest in the Δ*polB2*Δ*polB3* strain. On the other hand, the Δ*polB2* Δ*dbh* double mutant survived heat stress better than either single mutant.

### Spontaneous Mutation

With respect to its impact on the accuracy of chromosomal replication, deleting all three non-essential DNAPs was similar to inactivating only Dbh (PolY), as indicated by detailed comparison of spontaneous *pyrE* mutation spectra. Statistical tests characterized the set of *pyrE* mutations recovered in the triple-deletion mutant as being very similar to the corresponding mutations recovered in a *dbh* (*polY*) mutant. Most of the mutation sub-classes which differed in frequency among the three strains were those documented in previous studies of *S. acidocaldarius dbh* strains (Sakofsky et al., 2012); these properties were shared between the *dbh* (*polY*) single mutant CS2 and the triple polymerase mutant, which also lacks *dbh*. For two sub-classes, however (expansion of A tracts and G to A transition), removing both PolB2 and PolB3 had an apparent effect in the *dbh* (*polY*) background. In addition, for several other subclasses where *P*-values did not distinguish *dbh* strain from the triple mutant, they nevertheless did distinguish *dbh* from wild-type. The two subclasses of events that seemed to discriminate between *dbh* and triple mutant strains share no feature that seems to be mechanistically informative, however. We note also that these are the only two of 56 sub-classes of mutation we evaluated which met this criterion of low *P*-values for an apparent effect of PolB2 and PolB3, which raises the possibility that these low *P*-values may be a fortuitous result of these particular mutant samples. It should be feasible to resolve this question in the future with alternative mutation assays. We also note that in a different, yet related, species, deleting only the PolB2 gene (*dpo2*) had little impact on the spontaneous mutation spectrum of a different mutational target gene (Feng et al., 2020).

Measuring the incorporation and replication of DNA containing oxoG provided an independent test of a possible role of PolB2 and PolB3 in bypassing *oxoG* in the *S. acidocaldarius* chromosome. The results were consistent with those of the spontaneous mutation spectra, in that an obvious impact of PolB2 or PolB3 on oxoG bypass could not be detected, although it cannot be excluded that a subtle effect may have been masked by the impact of deleting *dbh* (*polY*).

## Conclusion

Given the diverse obstacles to DNA replication known to arise in cellular DNA, it seems likely that each of the three non-essential DNAPs of *S. acidocaldarius* may contribute in different ways to the growth and survival of cells, perhaps *via* multiple mechanisms. While the mechanisms remain to be defined in molecular terms, our analyses of mutants lacking these polymerases singly or in combination seem to support two generalizations. First, the limited consequences of removing all three non-essential DNAPs demonstrates that nearly all the DNA synthesis required by cells growing under laboratory conditions can be provided by the replicative DNAP PolB1. Second, the remaining two B-family DNAPs appear to have important but somewhat overlapping functions which become evident in the context of certain DNA-targeted stresses, especially when combined with functional Dbh. This hypothesis is motivated by the diverse treatments in which the Δ*polB2* Δ*polB3* construct was more sensitive than the single mutants or the triple-deletion construct, which provide new genetic evidence of the biological function for these two non-essential B-family DNAPs which are distributed widely among crenarchaea.

## Data Availability Statement

The original contributions presented in the study are included in the article/supplementary material, further inquiries can be directed to the corresponding author/s.

## Author Contributions

NK, SS, and DG conceived and designed the study. HM and RJ acquired the data and drafted the manuscript. HM, RJ, SS, DG, and NK analyzed and/or interpreted the data. NK and DG revised and completed the manuscript. All authors contributed to the article and approved the submitted version.

## Conflict of Interest

RJ was employed by the company ACGT, Inc., after concluding her participation in this research, and ACGT, Inc., played no role in conducting or reporting the research. The remaining authors declare that the research was conducted in the absence of any commercial or financial relationships that could be construed as a potential conflict of interest.

## Footnotes

1 https://www.eurofinsgenomics.jp/
